# The Boy to Succeed

**Published:** 1869-11

**Authors:** 


### THE BOY TO SUCCEED.

A few years ago, a large drug firm in this city advertised for a boy. Next day the store was thronged with applicants, among them a queer-looking little fellow, accompanied by a woman, who proved to be his aunt, in lieu of faithless parents, by whom he had been abandoned. Looking at this little waif, the merchant in the store promptly said: "Can't take him; places all full; besides he is too small." "I know he is small," said the woman, "but he is willing and faithful." There was a twinkle in the boy's eyes that made the merchant think again. A partner in the firm volunteered to remark that he "did not see what they wanted of such a boy—he wasn't bigger than a pint of cider." But after consultation the boy was set to work. A few days later a call was made on the boys in the store for some one to stay all night. The prompt response of the little fellow contrasted well with the reluctance of others. In the middle of the night the merchant looked in to see if all was right in the store, and presently discovered his youthful *protege* busy scissoring labels. "What are you doing?" said he. "I did not tell you to work nights." I know you did not tell me so, but I thought I might as well be doing something." In the morning the cashier got orders to "double that boy's wages, for *he is willing.*" Only a few weeks elapsed before a show of wild beasts passed through the streets, and, very naturally, all hands in the store rushed to witness the spectacle. A thief saw his opportunity, and entered at the rear door to seize something, but in a twinkling found himself firmly clutched by the diminutive clerk aforesaid, and after a struggle was captured. Not only was a robbery prevented, but valuable articles taken from other stores were recovered. When asked by the merchant why he staid behind to watch when all others quit their work, the reply was, "You told me never to leave the store when others were absent, and I thought I'd stay." Orders were immediately given once more: "Double that boy's wages; he is willing *and faithful.*" To-day that boy is getting a salary of $2,500, and next January will become a member of the firm. Young men imitate that example.

---

"Cider Vinegar" is a good antidyspeptic, and is often made of very injurious materials. Every household may protect itself from imposition by manufacturing its own vinegar, in the manner directed by the *Dubuque Northwestern Farmer.* "Soak common dried apples in water a few hours;" whether the writer means a barrel of dried apples in a pint of water as hot as melted iron, we cannot pretend to say; but, leaving the reader to find out the best way he can, in the course of several months' experiments, we proceed, remarking, in addition, that a "few hours" means two or three, or a thousand, according to circumstances. "While the apples are soaking, rub and wash them several times; remove the apples, strain the water through a tightly woven cloth; then, to a gallon of this water, add half a pint of molasses, and a piece of common brown paper," whether an inch or a mile square, we cannot say. Keep the jug, or vessel containing these ingredients, in the sun, or by a fire, and in a few days it will become cider vinegar fit for use, the apples not having been rendered unsuitable for cooking purposes by the process. While one jug is being used, another should be in preparation. The heat of the sun is preferable to that of the fire.

